# An Enhanced PSO-Based Clustering Energy Optimization Algorithm for Wireless Sensor Network

**DOI:** 10.1155/2016/8658760

**Published:** 2016-01-06

**Authors:** C. Vimalarani, R. Subramanian, S. N. Sivanandam

**Affiliations:** ^1^Department of Computer Science and Engineering, SNS College of Technology, Coimbatore 641 035, India; ^2^Department of Electrical and Electronics Engineering, SNS College of Technology, Coimbatore 641 035, India; ^3^Department of Computer Science and Engineering, Karpagam College of Engineering, Coimbatore 641 032, India

## Abstract

Wireless Sensor Network (WSN) is a network which formed with a maximum number of sensor nodes which are positioned in an application environment to monitor the physical entities in a target area, for example, temperature monitoring environment, water level, monitoring pressure, and health care, and various military applications. Mostly sensor nodes are equipped with self-supported battery power through which they can perform adequate operations and communication among neighboring nodes. Maximizing the lifetime of the Wireless Sensor networks, energy conservation measures are essential for improving the performance of WSNs. This paper proposes an Enhanced PSO-Based Clustering Energy Optimization (EPSO-CEO) algorithm for Wireless Sensor Network in which clustering and clustering head selection are done by using Particle Swarm Optimization (PSO) algorithm with respect to minimizing the power consumption in WSN. The performance metrics are evaluated and results are compared with competitive clustering algorithm to validate the reduction in energy consumption.

## 1. Introduction

Wireless Sensor Network is a network, which can self-organize them with a large number of small sensors. These sensor nodes can perform the packet transmission among themselves within their radio range and also they are organized in a way to sense, observe, and recognize the physical entity of the real world environment. WSN consists of a unlimited number of sensor nodes that can sense their vicinity and communicate either among themselves or to external base transceiver station. The best features of these wireless sensor nodes include small size, low cost, low computation power, multifunctional (can perform sensing, data processing, routing, etc.), and easy communication within short distances. In unattended hostile regions, these devices are deployed in general that make the power source of the sensors difficult to recharge. However, various research works and techniques are carried out for preserving energy in sensor nodes to extend the network lifetime [1]. Prolonged network lifetime, reliable data transfer, energy conservation in sensor nodes, and scalability are the main requirements for WSN applications. Because of the several constraints in the sensor nodes, WSN is having various issues such as coverage area, network lifetime, and scheduling and data aggregation.

**Figure 1:**
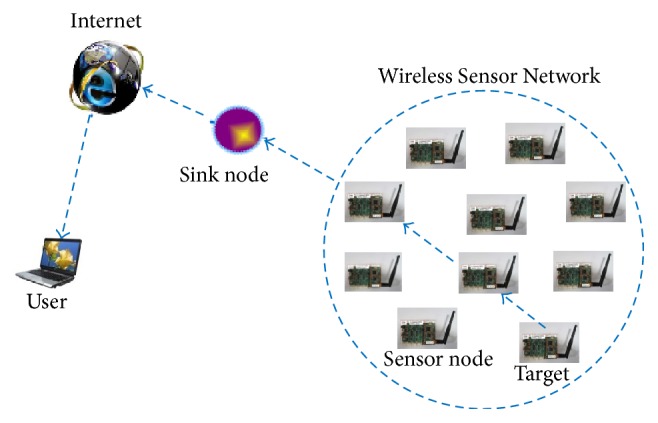
Architecture of Wireless Sensor Network.

The architecture of WSN shows in Figure 1; it comprises wireless sensor nodes in huge number which has been arranged and installed based on the applications and a sink or base station (BT) that is located very near to or within the radio range. The BT transmits the queries to the neighboring sensor nodes which perform the sensing task and return the data to the BT as an answer to the transmitted query.

In WSN nodes utilize disproportionate amount of energy for communication and the required energy in terms of battery power to transmit the packet will differ among the transmissions with respect to the distance between the sender and receiver nodes; therefore multihop communication is recommended. Data transmission using hierarchical routing which increases the lifetime of the sensor network by grouping a number of nodes into clusters. Then a head node is selected for each cluster known as cluster head to collect the data from its members and transmit to the base station with a minimum cost of multihop transmission.

Most of the algorithms and protocols [2, 3] tried their best to enhance the performance and throughput of the networks, such algorithms are Low-Energy Adaptive Clustering Hierarchy (LEACH), a Hybrid Energy Distributed Clustering Approach (HEED), and so on. Earlier research papers [4–8] provide different energy efficient techniques that can be used at network layer level by using different routing protocols with energy efficient routing algorithms and reliable communications. The mechanisms described in these algorithms relatively increase the utilization of the power in packet transmission and lengthen the life of the sensor networks. The proposed research work implements the PSO in clustering and for optimal selection of cluster head to enhance the improvement in the residual energy of node by sending a data packet to the cluster head which is located very nearest to the BT.

Particle Swarm Optimization (PSO) is an optimization technique in which natural species social behaviors are considered for the purpose of computation [9]. It is a swarm intelligence technique which is based on population that performs optimization process with the objective of optimizing a fitness function. This approach makes use of a swarm for the purpose of search on every particle and records the fitness value of each particle. Then the particles are linked with their matching velocity. It will help the particle to make a move to a proper location by considering the optimized fitness function's cost [10, 11]. From all the particles of intelligence local, best position optimizes the global best position to identify the cluster head position in order to minimize the overall energy consumption. PSO algorithm has more efficiency and throughput when compared with other mathematical and heuristic approaches.

### 1.1. Rationale of the Work

To enhance the network lifetime appropriately many routing protocols and cluster-based algorithms are used to fulfill the application requirements in WSN. From existing research methods, optimizing energy dissipation for communication becomes very critical. For maximizing lifetime of the WSN, part of an energy consumption of each sensor node has an important role while communicating among other sensor nodes. This research work focuses on energy conservation in each sensor node by using PSO-based clustering and cluster head selection energy optimization algorithm. The cluster head is selected using PSO, based on the distance from the cluster member node to sink node (BT) and the residual energy in that node. Simulation results show that the motivation of this work improves the life expectancy of the network significantly.

## 2. Related Work

WSNs have many research challenges and network issues when deploying the sensor nodes to monitor the physical world. Hierarchical routing protocols are appropriate for organizing the nodes to increase the scalability of the WSNs. The traditional clustering algorithm LEACH [12] uses randomized rotation with uniform clustering of local cluster heads to increase the scalability and network performance. The lifetime of the network has extended by utilizing a HEED clustering protocol [13]; this formed the clustering and cluster head selection based on the residual energy of sensor nodes and the cost of communication from source to destination.

Paper [14] proposed Energy Efficient Hierarchical Clustering (EEHC) that increases the lifetime of the sensor network. However, hierarchical clustering made overload in cluster heads and reduces its power sooner than the other nodes. Paper [15] proposed a distribution scheme of cluster heads to reduce energy dissipation by avoiding unnecessary redundancy and compared with existing LEACH it prolongs network lifetime. Paper [16] proposed energy efficient adaptive multipath routing technique to reduce routing overhead and efficiently utilizes the energy availability. In paper [17] competitive clustering (CC) algorithm with sink mobility was proposed to increase residual energy in sensor nodes and improve the network performance. It selects the final head among the competitive candidates based on their remaining energy and competition radio range length. This algorithm forms clusters in small size near the fixed sink node that makes the head node be closer to the BS and consumes lower energy during data gathering between the clusters.

For implementing individual sensor nodes in WSN the better optimization approaches which requires reasonable memory space and resources to produce better results [18]. One of the popular optimization techniques is called Particle Swarm Optimization that has the advantage of solutions with better quality, higher efficiency in computation, easy implementation, and high speed of convergence. PSO-clustering in [14] handled NP-hard optimization problem efficiently by using clustering based on a cluster which lies in a nearby neighborhood, and choosing the sensor node closer to base station becomes header for that particular cluster. PSO-C algorithm considers available energy and distance between the nodes with respect to their cluster heads [19]. The authors in [20] have showed that PSO outperforms both LEACH and LEACH-C in terms of the network span and the overall throughput.

In [21] the author proposed graph theory for routing and PSO for multihop sensor network. For each *i*th round, the cluster head is selected with the help of a weighted function denoted as *w*(*i*), which will be computed in an iterative manner. Based on the distance taken by the data packet to reach a destination node from the source node and remaining energy, routing of packets is optimized with the fitness function. The simulation results are evaluated with the competitive clustering approach of electing cluster heads and shown as positive results. With the goal of maximizing the network coverage in mobile sensor networks, the author in [22] applied PSO to optimize the sensor deployment strategy. It is executed in a centralized manner which increases the burden of the BS.

For the purpose of reducing the intracluster distance, the authors of paper [23] proposed PSO-based cluster head selection approach to identify the best locality for head nodes with an aim to localize the center of cluster density. Simulation results are matched with the existing LEACH-C and PSO-C and an improvement in network lifetime and saving energy is shown. The recluster construction made network overhead and additional power consumption for communicating clustering information from base station to the sensor nodes.

In this paper, an enhanced clustering algorithm using PSO technique is proposed for energy conservation. The optimal selection of cluster head using PSO reduces the power consumption of each sensor nodes by sending data packets to its cluster head instead of directly forwarding to the base station.

## 3. Network Energy Model

In this paper, the proposed work simulates the WSN consisting of “*n*” number of sensor nodes deployed for a temperature monitoring applications using rectangular sensor network. Some assumptions are made regarding the deployment of nodes as [12] given in the following:

- All the chosen nodes are considered as static after deployment.
- Two types of nodes are as follows: one is sensor node for sensing temperature monitoring environment and another type of node is sink or base station fixed in the center of the sensor network.
- Sensor nodes are assigned with a distinctive identification (ID) and similar preliminary energy.
- Node is allowed to use transmission power with different levels which are preferred to the remoteness to the target node.
- The BT once in a while sends a request message in terms of the packet to the cluster head for getting sampling data from sensors.
- Links are symmetric.

### 3.1. Energy Model

In WSN an energy model designed in physical layer discussed in [12] used for calculating energy loss in each sensor node while communicating with other sensor nodes. Two channel propagation models used are the free space (*d* ^2^ power loss) for the purpose of one-hop or direct transmission and the multipath fading channel (*d* ^4^ power loss) for packet transmission via multihop. Thus, the energy exhausted for this kind of transmission of an *l*-bit packet over distanced *d* is calculated as

(1) $$\begin{array}{c} E_{TX}\left(\;l,d\;\right)=\left\{\begin{array}{cc} lE_{elec}+l\varepsilon_{fs}d^{2}, & d<d_{0},\\ lE_{elec}+l\varepsilon_{mp}d^{4}, & d\geq d_{0}, \end{array}\right. \end{array}$$

where *ε*_fs_ is free space energy loss, *ε*_mp_ is multipath energy loss, *d* is distance between source node and destination node, and *d*_0_ is crossover distance:

(2) $$\begin{array}{c} d_{0}=\sqrt{\frac{\varepsilon_{fs}}{\varepsilon_{mp}}}. \end{array}$$

The energy spent for the radio to receive this message is

(3) $$\begin{array}{c} E_{RX}\left(\;l\;\right)=lE_{elec}. \end{array}$$

Thus the transmission power and receiving power energy levels are designed in physical and Mac layer of the WSN [21].

## 4. Proposed PSO-Based Clustering Algorithm

In this section, we propose an Enhanced PSO-Based Clustering Energy Optimization (EPSO-CEO) algorithm to form clusters and cluster head selection with a combination of centralized and distributed method using static sink node.

### 4.1. Particle Swarm Optimization (PSO)

Particle Swarm Optimization (PSO) is a population-based optimization scheme. The random solutions of the system are initialized with a population and search optimal solutions in each generation [20]. The potential solutions in each generation are called particles. Each particle in PSO keeps the stored record for all its coordinates which are related to obtaining the better solution by following the current best particles.

Fitness function of every particle is executed and the fitness value (best solution) is calculated and stored. The fitness value of the current optimum particle is called “pbest.” PSO optimizes the best population value that is obtained so far by any particle in the neighbors and its location is called lbest.

**Figure 2:**
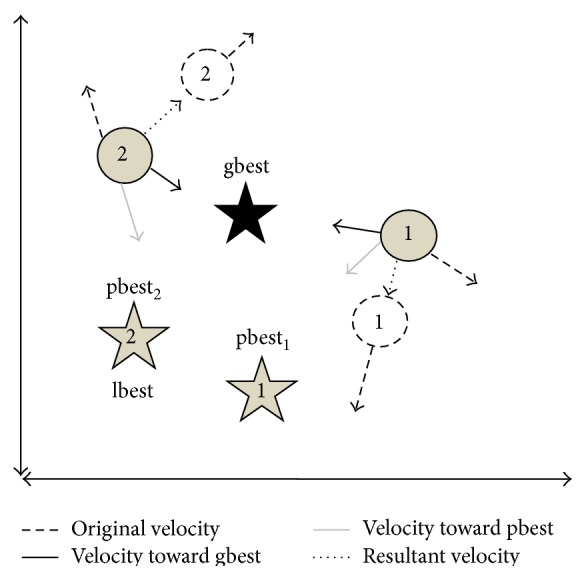
PSO particle movements.

When all the generated populations are considered as topological neighbors by a particular particle, then the best value is chosen among the generated population and that particular best value is the best solution and it is known as gbest.  Figure 2 shows the PSO particle movement in a two-dimensional space.

The PSO always try to change the velocity of every particle towards its pbest and lbest. The velocity is determined by random terminologies, which is having randomly generated numbers for velocity towards pbest and lbest localities.

From the large deposit of generated solutions, the best one is selected to resolve the problem. The PSO algorithm always stores and maintains a record of results for three global variables such as target value or condition, gbest, and stopping value.

Every obtained particle of PSO contains the following details.

- A data which can represent a global solution.
- Value for velocity which will indicate the amount of data to be changed.
- lbest value.

### 4.2. Cluster Formation

The cluster is formed by the base station or sink on the basis of centralized clustering. For clustering base station (sink) broadcasts info collection message to all sensor nodes. Sensor nodes after receiving this message start to send its node information such as node id, location (distance from the base station in *X*  and  *Y* position), energy loss and energy loss ratio (velocity), and current energy to send base station. Then base station initiates the clustering process steps as follows.

**Step 1 .**

Conversion of problem into the PSO space in which the PSO particle has two dimensions such as particle position and velocity.

**Step 2 .**

Estimation of fitness value using fitness function.

### 4.3. Fitness Function

**Figure 3:**
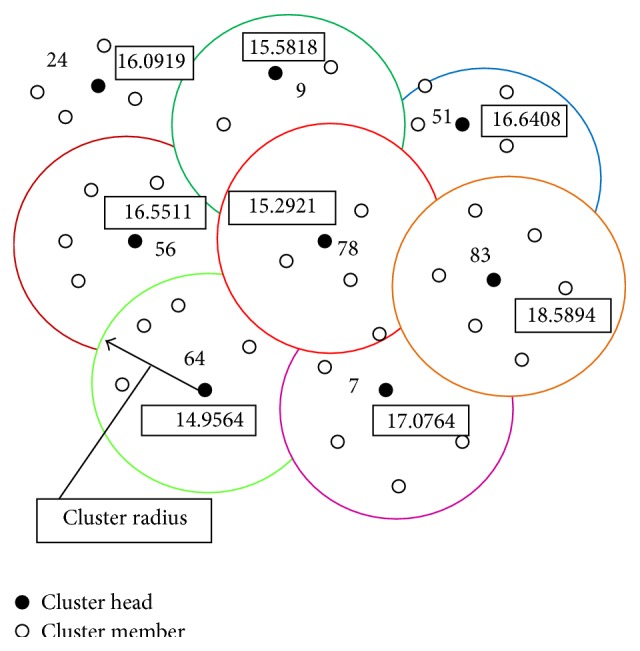
Cluster formation.

Our proposed fitness function for PSO-based clustering is to optimize the average distance and average energy of the member nodes and from the current cluster head and headcount.  Figure 3 shows the cluster formation using PSO.

The fitness value is calculated for the particle by using the formula given in the following:

(4) Fitness  value=Fv=α1·∑i=0ndcurrent  node,member  in+α2·∑i=0nEmember  iEcurrent  node+1−α1−α2·1No  of  members  covered  by  current  node,

where *α*_1_ and *α*_2_ are weighing parameters (normalized values) and *n* denotes number of members covered within the cluster.

**Step 3 .**

Generation of new particles from the initial solution. Formation of new particles from the old one is a generation of a new particle.

*Step 3.1*. Estimation of new velocity: the current velocity of a taken particle is considered to the rate at which the particle's position is changed. New velocity is calculated as follows:

(5) new  velocity=ω∗old  velocity+w1local  best  position−current  best  position+w2global  best  position−current  best  position,

where *ω* is inertia weight and *w*_1_ and *w*_2_ are basic PSO tuning parameters.

*Step 3.2*. Estimation of new position of the particle is as follows:

(6) $$\begin{array}{c} \text{new position}=\text{old position}+\text{new velocity}. \end{array}$$

Finally the new particle (new velocity and new position) arrives.

**Step 4 .**

Calculation of fitness value for new particles.

Fitness value of the new particles is estimated by using fitness function in Step 2 with new velocity and new position.

**Step 5 .**

Fitness value of old particle and new particle is compared and the best one is selected for the next iteration:

 If new fitness value > old fitness value
 select new particle;
 else
 old particle is forwarded to next iteration.

**Step 6 .**

For every iteration, one best solution is selected as a local best solution.

The particle which has maximum fitness value in the current iteration is selected as lbest solution.

**Step 7 .**

The local best solutions from all iterations of the particle in which has maximum among all solutions are selected as a global best solution. The final solutions are decoded into clusters.

The base station forms the cluster using PSO and broadcasts a cluster-announcement message to sensor nodes which contains cluster information as shown in Figure 3. Each sensor node stores this message and initiates round procedure to perform cluster head selection.

### 4.4. Cluster Head Selection

**Figure 4:**
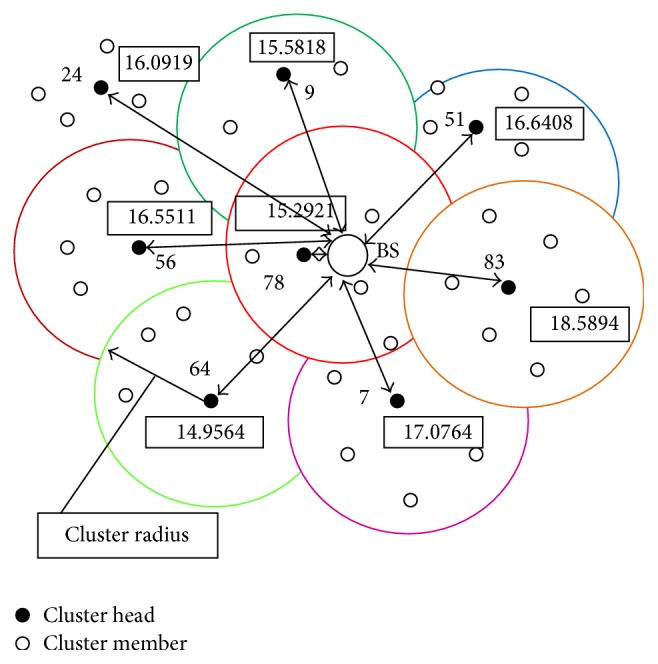
Cluster head selection.

After clustering, each sensor node maintains “my_cluster_list.” It includes current cluster-id, velocity, location, and energy. Then the round procedure is initiated to perform cluster head selection. Cluster head selection by implementing PSO algorithm is shown in Figure 4.

**Step 1 .**

The members that are covered by the current node are communicated with each other to select a cluster head which follows as steps mentioned below.

**Step 2 .**

Fitness function:

(7) Fitness  value=Fv=α1·∑i=0mdcurrent  node,member  in ϒ+ α2·∑i=0mEmember  iEcurrent  node ϒ+1−α1−α2·1No  of  members  covered  by  current  node,

where $ϒ=\left\{\begin{array}{cc} 1, & if\;member\;i\;is\;covered\;by\;current\;node\\ 0, & else \end{array}\right\}$, *m* is number of members in the current cluster node, *α*_1_ and *α*_2_ are weighing parameters (normalized values), and *n* denotes the number of members covered within the competition range.

**Step 3 .**

Generation of new particles from the initial solution.

*Step 3.1*. Estimation of new velocity is as follows:

(8) new  velocity=ω∗old  velocity+w1local  best  position−current  best  position+w2global  best  position−current  best  position,

where *ω* is inertia weight and *w*_1_ and *w*_2_ are the basic PSO tuning parameters.

*Step 3.2*. Estimation of new position by using new velocity is as follows:

 New position = old position + new velocity.
 Finally the new particle (new velocity and new position) arrives.

**Step 4 .**

Calculate fitness value of new particles.

Fitness value of the new particles is estimated by using fitness function given in Step 2 with new velocity and new position.

**Step 5 .**

Fitness value of old particle and the new particle is compared and the best one is selected for the next iteration:

 if new fitness value > old fitness value
 select new particle;
 else
 old particle is forwarded to next iteration.

**Step 6 .**

For every iteration, one best solution is selected as a local best solution.

The particle which has maximum fitness value in the current iteration is selected as lbest solution.

**Step 7 .**

In all iterations one local best solution is found and the particle which has maximum among all local best solutions is selected as a global best solution. Finally, the particle which has a global best solution is chosen as a current cluster head as shown in Figure 4.

### 4.5. Data Transmission Using Multihop Routing Protocol

#### 4.5.1. Intracluster Multihop Routing Setup

After clustering, routing procedure is invoked during data transmission. Routing consists of two steps; one is route establishment and another one is forwarding sensed data. On demand distance vector routing protocol is used for route establishment [24] between sensor nodes in two occasions: (1) initial route establishment and (2) route unavailability.

In route establishment phase route request message is broadcasted to all nodes with one-hop transmission [20] and unicast route reply message is sent in reverse path to the source node. Once the route is established data transmission with the multihop routing protocol is commenced.

In this work, the multihop communication protocol is used for data transmission [5–8] between the nodes to cluster head (intracluster routing) and cluster head to BT (destination). Data aggregation is done by the head in each cluster for the purpose of saving the residual energy and setting up the threshold value *d* threshold. In the case of the transmission distance between the cluster head node and the base station is smaller than the threshold value; then the cluster head is committed to transmit the calculated aggregated data to the head with the single hop transmission. Otherwise, cluster head will find next hop with minimum cost neighbor as a relay node [12]. Also, this node will be chosen based on the distance and residual energy. The minimum cost path and highest residual energy node is calculated by using the formula as given in the following:

(9) Costj=ω∗∗dsi,sj2+dsj,SN2max⁡dsi,sj2+dsj,SN2+1−ω∗max⁡Ej−Ejmax⁡Ej,ω∈0,1,

where *ω* is randomized tuning parameter, *s*_*i*_, *s*_*j*_ are the member node and current head node, and SN denotes sink node.

Relay cluster head node is selected with minimum cost to send data to a destination and intercluster routing is established once the cluster head is selected.

## 5. Results and Discussion 

### 5.1. Simulation Results

The Network Simulator (NS-2.34) is used for designing the network scenario which executes the PSO algorithm to form clusters and selecting cluster heads in order to reduce energy conservation of sensor nodes. Simulation results are produced, by deploying 100 nodes within a 200*∗*200 Sqm area. The sensor nodes are deployed with the task of sensing physical parameters.

The simulation results are evaluated in terms of the following performance metrics.

*Total Number of Packets Received*. The total number of data packets received by sink node calculated the count value of the total number of data packets transmitted by cluster head node and received by sink node (base station).

*Packet Delivery Ratio*. The number of packets successfully received with respect to the total number of packets transmitted is known as packet delivery ratio.

*Normalized Overhead (NRO)*. The normalized overhead is defined as the computed ratio between the number of control packets and the number of data packets.

*End-to-End Delay*. The average time taken to route a data packet from source node to target node is calculated as delay in seconds.

*Throughput*. It is a measure of a number of packets transmitted per second.

*Number of Packet Drop*. It is a difference between the total number of data packets sent and the total number of data packets received.

*Packet Dropping Ratio*. It is a ratio between number of packets dropped and number of packets transmitted.

*Network Lifetime*. This metric evaluates the time at which the first node failure occurs due to the discharge of battery power charge. The number of active nodes in each round is depicted in

*Relative Energy Consumption*. It gives the ratio of total amount of energy consumed and the transmission of total packets.

*Total Energy Consumption*. This calculates the total amount of energy consumed by the nodes to transfer the packets through the simulation.

*Average Energy Consumption*. It gives the relation of the total energy consumed for total packets received and the total amount of energy consumed by the nodes to transfer the packets.

*Total Residual Energy*. It is the difference of the initial energy and current energy of each sensor node.

*Average Residual Energy*. It is the overall residual energy of all sensor nodes over total simulation time.

The network simulation parameters and PSO parameters are listed in the following.

### 5.2. Simulation Parameters

Simulation parameters are as follows:

 Number of iterations: 100.
 Number of nodes: 100 nodes.
 Area (deployment): 200*∗*200 Sqm.
 Initial energy: 3 joules.
 Energy indulgence to run the radio device (*E*_elec_): 50 n joule/bit.
 Coverage area: 91 metre^2^.
 MAC type: 802.11.
 Antenna model: Omni Direction Antenna.
 Propagation model: free space/two-ray ground.
 Queue type: priority queue.
 Transmission power: 0.02 watts.
 Receiving power: 0.01 watts.
 Application type: sensing application (temperature).
 Connection type: UDP.
 Transmission duration: 155 seconds.
 Simulation time: 200 sec.

### 5.3. Performance Analysis

In this research work, the following performance metrics are taken for evaluating the WSN to enhance the network efficiency by saving energy. Metrics are evaluated for various rounds from 25, 50, and 75 to 200 and the corresponding outputs depicted as a graph (Figures 5–17). Tables 1 and 2 show the evaluation of the simulation results acquired by using competitive clustering algorithm and PSO-based cluster head selection algorithm. Tables 1 and 2 provide numerical data with a slab of 50 from 50 to 200 rounds.

Simulation results are evaluated with respect to the performance metrics such as number of packets received by sink node, end-to-end delay, packet drop in terms of number of packets, packet dropping ratio, packet delivery ratio, normalized overhead, and throughput of the proposed PSO-based clustering algorithm in contrast with the competitive clustering algorithm that is shown in Table 1.

The other metrics such as overall residual energy, average energy consumption, relative energy consumption, average residual energy, total energy consumption, and lifetime are compared and results are given in Table 2.

From these tables, we observed that the overall network performance of WSN is increased by enhancing the clustering algorithm using PSO-based cluster head selection scheme. Specifically, the average energy consumption and total energy utilization are reduced by 40% and the lifetime has been enhanced by 70%.

The various performance metrics with reference to the graph are discussed as follows.

**Figure 5:**
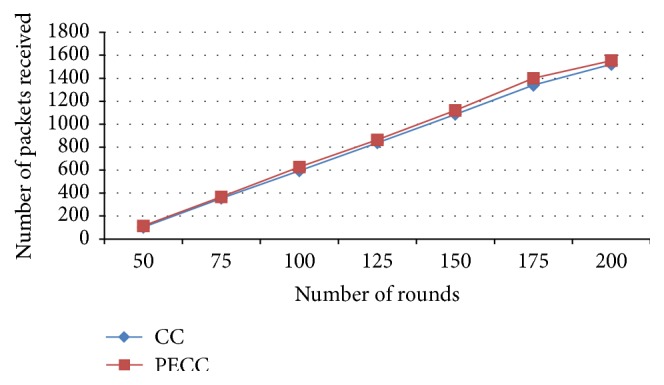
Packets received.

Figure 5 shows the number of data packets received by the BT with varying rounds from 50, 75, 100, 125, 150, and 175 to 200, respectively. From the results, it can be seen that number of packets received is increased with the optimal selection of cluster head scheme by using proposed PSO-clustering algorithm compared to the competitive clustering algorithm.

**Figure 6:**
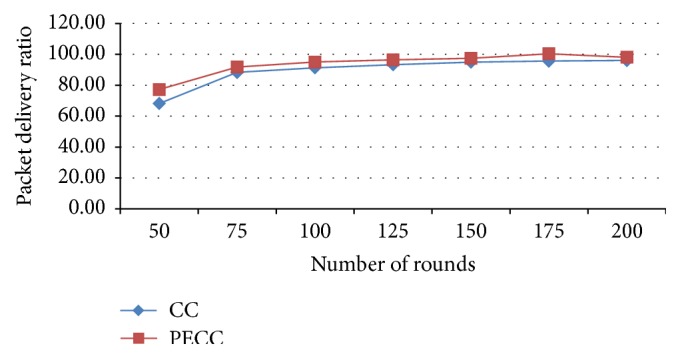
Packet delivery ratio.

Figure 6 shows packet delivery ratio that obtained by using PSO-based energy optimization algorithm is considerably increased compared with the existing system.

**Figure 7:**
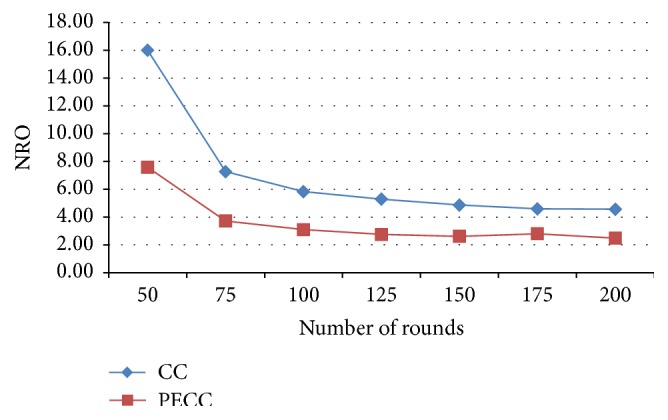
Normalized overhead.

Figure 7 depicts the comparison of the normalized overhead using competitive clustering and enhanced PSO clustering algorithm. In proposed system, the number of control packets and normalized overhead of the whole network transmission are reduced considerably. Normalized overhead of the overall network control packets is reduced with respect to increased data packets.

**Figure 8:**
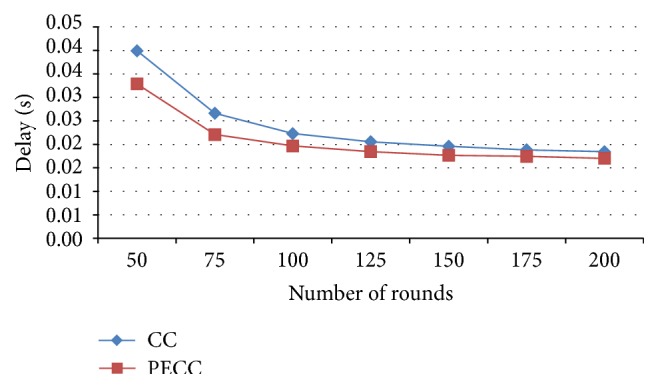
End-to-end delay.

Figure 8 shows end-to-end delay of the PSO-based energy algorithm which is minimum compared with a competitive clustering algorithm.

**Figure 9:**
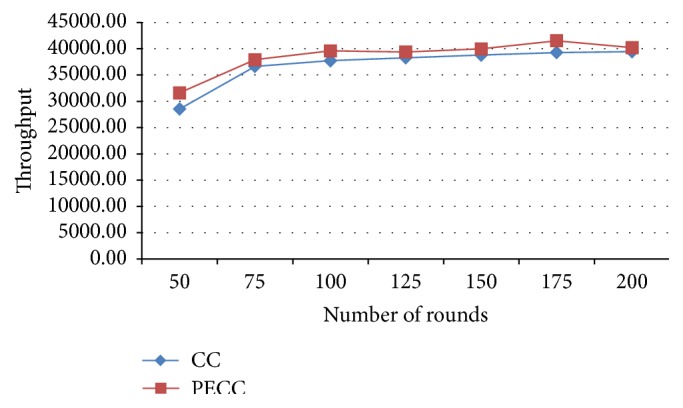
Throughput.

Figure 9 shows an improvement of throughput in varying rounds compared with existing algorithm.

**Figure 10:**
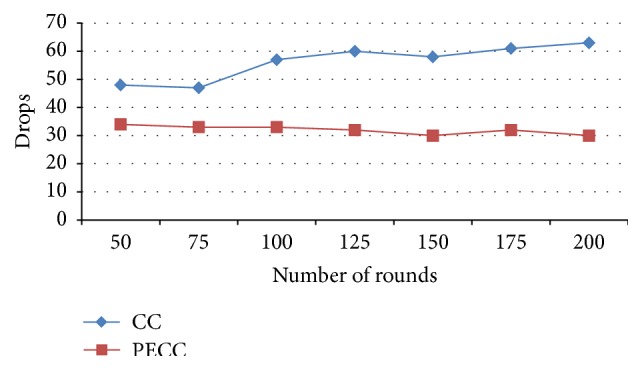
Number of packet drops.

In Figure 10 the number of packets dropped in overall packet transmission of network using PSO clustering and the competitive clustering algorithm is compared. It is observed that the packet drops are reduced considerably.

**Figure 11:**
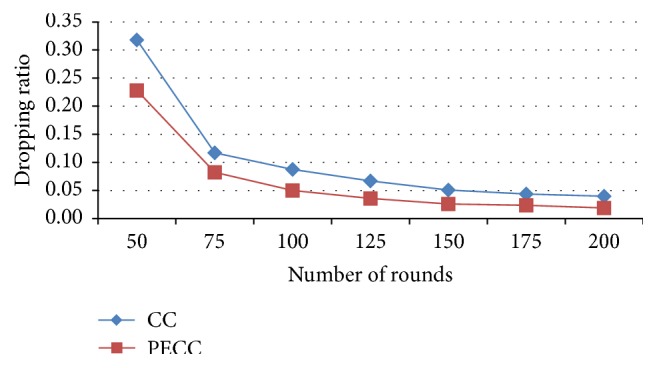
Dropping ratio.

The dropping ratio is reduced in proposed PSO clustering algorithm relatively in comparison with competitive clustering algorithm which is shown in Figure 11.

**Figure 12:**
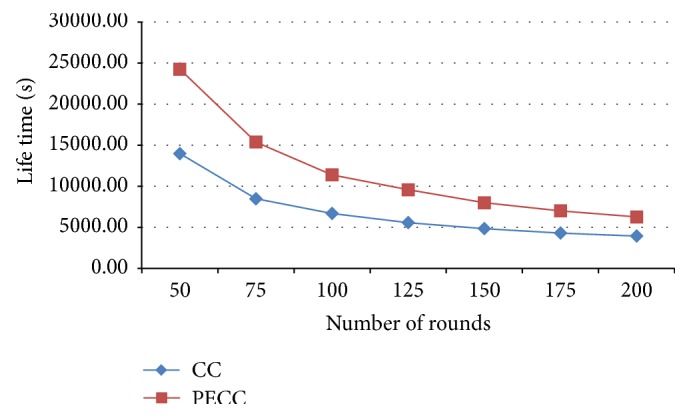
Lifetime.

Figure 12 which shows the lifetime of the network is increased by consuming less energy of each sensor node with a selection of optimal cluster head using proposed PSO compared with the competitive clustering. The number of active nodes in each round which shows the lifetime of the network is increased.

**Figure 13:**
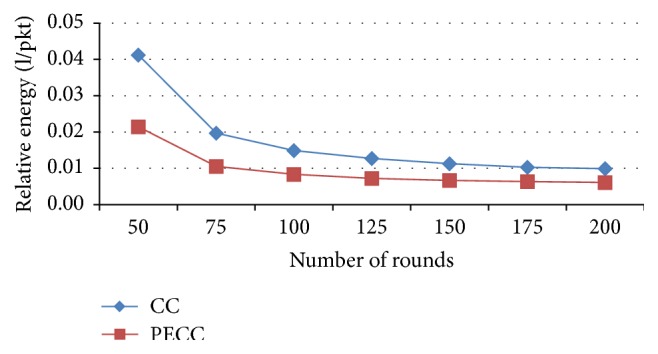
Relative energy.

Figure 13 shows that the relative energy consumption of proposed PSO clustering is less than competitive clustering.

**Figure 14:**
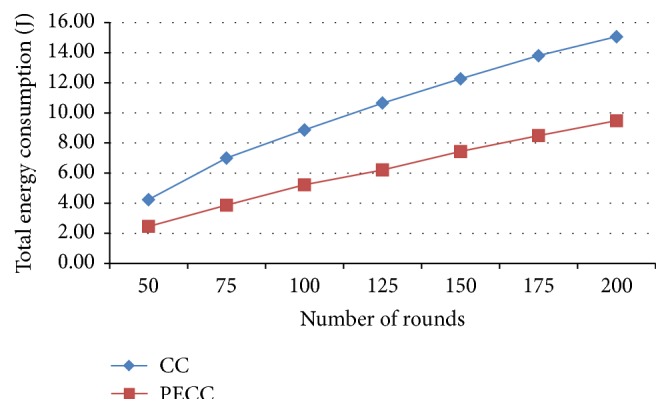
Total energy consumption.

In Figure 14, it is observed that the total energy consumption for various rounds to transmit packets by using proposed PSO-clustering is decreased when compared with competitive clustering considerably.

**Figure 15:**
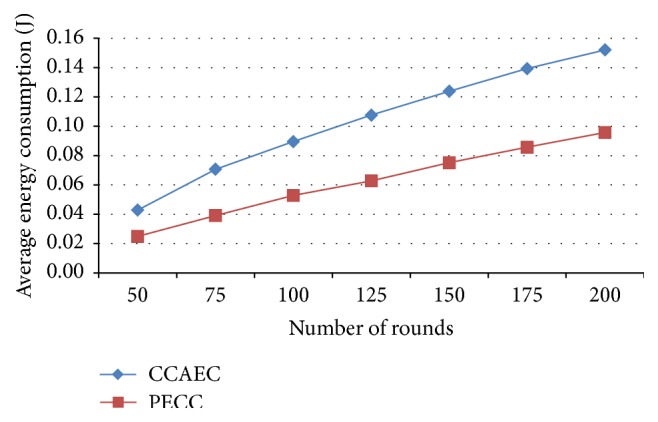
Average energy consumption.

In Figure 15 it is observed that the energy consumption of all nodes is less considerable by using proposed PSO clustering than competitive clustering.

**Figure 16:**
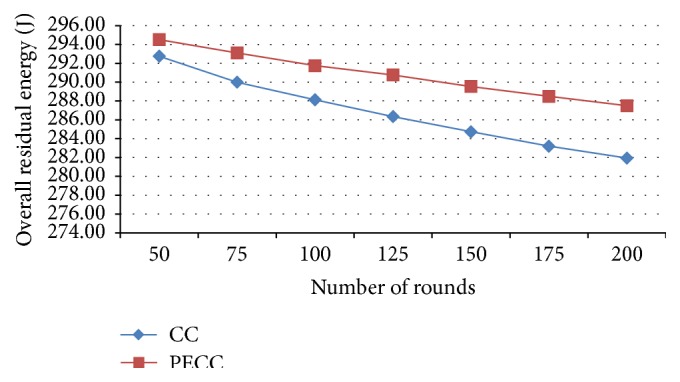
Overall residual energy.

A considerable improvement is observed in overall residual energy in proposed PSO clustering when compared to the competitive clustering which is shown in Figure 16.

**Figure 17:**
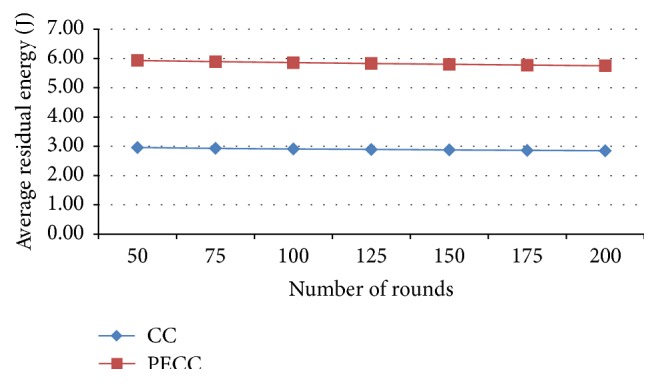
Average residual energy.

Figure 17 shows that the average residual energy is increased in proposed PSO clustering compared with the competitive clustering.

From the simulation results and performance analysis, we observed that the optimal selection of cluster head using PSO clustering algorithm is energy efficient in terms of saving energy and increases the network lifetime to improve the performance of WSNs.

## 6. Conclusions

The network performance of the WSNs is enhanced by various PSO-based clustering and cluster head selection scheme algorithms in terms of increasing the throughput, packet delivery ratio, residual energy, and number of active nodes. The enhanced PSO algorithm constructs clusters in a centralized manner within a base station and the cluster heads are selected by using PSO in distributed manner. The sensed data from the sensor nodes are aggregated by the head and transmit to the BT directly or using relay node based on the threshold value for which the multihop routing protocol is used. The performance metrics such as throughput, packet delivery ratio, network lifetime, normalized overhead, delay, residual energy, and total energy consumption are evaluated and compared with competitive clustering methodology. The simulation outcome shows that the projected (ECPSO-CEO) scheme gives improved performance in order to minimize the total consumed energy and increase the lifetime of WSN. In future, this work can be extending to improve the network lifetime and data transmission using multiple sink or mobile sink [25] and efficient data collection using data aggregation [6] owing to reduction of the delay in a certain level in the proposed system.

### 6.1. Contribution to Knowledge

Our research work focuses on energy conservation in each sensor node by using PSO-based clustering and cluster head selection energy optimization algorithm. The cluster head is selected using PSO, based on the distance from the cluster member node to sink node (BT) and the residual energy in that node. To increase the lifetime of the WSN energy conservation measures and energy optimization techniques are enhanced.

## Figures and Tables

**Table 1:** Comparison of simulation results of proposed PSO-based clustering algorithm and competitive clustering algorithm.

Number of rounds		50	100	150	200
Number of packets received	CC	103	595	1086	1521
	PSO-based	115	627	1120	1554

Delay (s)	CC	0.04	0.02	0.02	0.02
	PSO-based	0.03	0.02	0.02	0.02

Drops	CC	48	57	58	63
	PSO-based	34	33	30	30

Dropping ratio (%)	CC	0.32	0.09	0.05	0.04
	PSO-based	0.23	0.05	0.03	0.02

PDR (%)	CC	68.21	91.26	94.93	96.02
	PSO-based	77.18	95	97.39	98.11

NRO (%)	CC	16.00	5.83	4.87	4.56
	PSO-based	7.58	3.1	2.62	2.48

Throughput (b/s)	CC	28541.60	37724.50	38789.50	39438.90
	PSO-based	31605.4	39597.8	39977.9	40200.5

**Table 2:** Comparison of simulation results of proposed PSO-based clustering algorithm and competitive clustering algorithm.

Number of rounds		50	100	150	200
Overall residual energy (J)	CC	292.75	288.13	284.73	281.94
	PSO-based	294.52	291.75	289.54	287.5

Average energy consumption (J)	CC	0.04	0.09	0.12	0.15
	PSO-based	0.02	0.05	0.08	0.1

Average residual energy (J)	CC	2.96	2.91	2.88	2.85
	PSO-based	2.97	2.95	2.92	2.9

Relative energy in (l/pkt)	CC	0.04	0.01	0.01	0.01
	PSO-based	0.02	0.01	0.01	0.01

Total energy consumption (J)	CC	4.25	8.87	12.27	15.06
	PSO-based	2.47	5.23	7.44	9.48

Lifetime (s)	CC	13983.80	6697.76	4842.70	3944.72
	PSO-based	24252.1	11393	7998.28	6273.96
